# Grounding of abstract concepts related to power

**DOI:** 10.3758/s13421-023-01492-6

**Published:** 2023-12-11

**Authors:** Martina Rieger, Victoria K. E. Bart

**Affiliations:** Institute of Psychology, Department of Psychology and Sports Medicine, UMIT TIROL - Private University for Health Sciences and Health Technology, Eduard Wallnöfer Zentrum 1, 6060 Hall in Tirol, Austria

**Keywords:** Grounded cognition, Abstract concepts, Power, Drawing

## Abstract

Grounded cognition assumes that language and concepts are understood using simulations in different modalities. Evidence for this assumption mainly stems from studies using concrete concepts. Less evidence for grounding exists for abstract concepts, which are assumed to be grounded via metaphors associated with them or via experiences with them in specific situations. In the present study, we developed a new paradigm and investigated grounding of abstract concepts related to power or the exercise of power. As stimulus material, we chose pairs of concepts, for example, democracy and dictatorship. Participants were presented each concept separately and were asked to create a visual image in their mind. Then they were asked to rate images on several aspects. Afterwards they were asked to draw a sketch of the image. Results showed that drawings of high-power concepts had a larger vertical extension than low-power concepts. Results of the questions depended on the specific concepts. For instance, wealth (high-power) was rated as more colorful than poverty (low-power), but democracy (low-power) was rated as more colorful than dictatorship (high-power). These results may partly be explained by the valence of the concepts. Drawings often contained persons, objects, and situations, but were rarely abstract. Sometimes drawings contained metaphorical content and sometimes the content of drawings related to specific experiences. In conclusion, abstract concepts related to power can be depicted visually via grounding in different ways, such as using metaphors, experiences, and actions.

**Supplementary Information:**

The online version contains supplementary material available at 10.3758/s13421-023-01492-6.

## Introduction

Grounded cognition theories assume that cognition is grounded in modal systems. Whereas it seems intuitive that concrete concepts are grounded in modal systems, one major criticism of grounded cognition is often that it is difficult to explain how abstract concepts, which lack a physical referent, are grounded (Mahon & Caramazza, 2008; Mahoon, 2015). However, research has indicated several ways in which grounding of abstract concepts may take place (e.g., Conca et al., 2021; Harpaintner et al., 2022). In the present study, we employed a new paradigm, asking participants to think of visual images of abstract concepts and to draw a sketch of them. We used abstract concepts related to power. It has previously been shown that power is associated with the vertical dimension of space (Schubert, 2005). We were interested whether an association between relative power and the vertical dimension of space is observable in drawings. Further, we were interested in participants’ ability to depict abstract concepts visually and in the content of visual depictions to investigate the way in which they are grounded.

Traditionally, cognitive concepts are viewed as abstract, amodal mental entities that are independent from modal systems such as those of perception and action (e.g., Pylyshyn, 1984). Modal information of objects and events is transformed into an amodal representational format and cognitive processes work with those amodal representations. In contrast, grounded cognition theories argue that cognition is grounded in modal systems for perception (e.g., vision, hearing), action (e.g., movement, proprioception), and introspection (perception, monitoring, and use of internal states such as affects, motivation, intention, and metacognition) (e.g., Barsalou, 2008). Further, grounding of cognition in the physical and social environment is emphasized (Barsalou, 2003; Kiefer & Barsalou, 2013). Accordingly, cognition consists of multiple multimodal simulations (perceptual symbols theory and situated action account; Barsalou 1999, 2008, 2009).

Assumptions of grounded cognition may not only apply to concrete concepts (e.g., flower) but also to abstract concepts (e.g., democracy; Barsalou, 2003; Barsalou, 2008; Kiefer & Barsalou, 2013; for an overview see Kiefer & Pulvermüller, 2012). For abstract concepts no direct sensory-motor experience is available, because they lack a physical referent (e.g., Paivio, 1986). Thus, it is often thought that abstract concepts require amodal representations (Mahon & Caramazza, 2008; Mahoon, 2015). However, empirical evidence shows that when participants are asked to generate properties of abstract concepts, i.e., to report features, situations, and associations referring to the abstract concept (Barsalou & Wiemer-Hastings, 2005; Harpaintner et al., 2018), abstract concepts are associated with multimodal features (Harpaintner et al., 2018), which differ in their relative dominance depending on the abstract concept. In some abstract concepts social constellations (e.g., argument), in others sensorimotor features (e.g., insight), in others still verbal associations (e.g., dignity) dominate (Harpaintner et al., 2018). Evidence for this comes not only from behavioral but also from neuroimaging studies (see Kiefer & Harpaintner, 2020, for a review). Thus, abstract concepts are not exclusively based on linguistic or amodal content, but are, like concrete concepts, grounded in multimodal representations.

Another popular idea is, that, instead of being grounded in multimodal representations, abstract concepts are grounded via metaphors (theory of metaphor, Lakoff & Johnson 1980a, b). Abstract concepts are usually defined in reference to several concrete concepts (relating to space, movement, food, or objects) in different metaphors (Lakoff & Johnson 1980a). Metaphors develop through physical and cultural experience in interaction with the environment (Lakoff & Johnson, 1980a). Evidence indicates that people do indeed sometimes refer to metaphors when they talk about abstract concepts and, further, that how they speak about abstract concepts differs depending on culture (Casasanto et al., 2004).

One abstract concept that has received some attention is the concept of power. Power is grounded in the vertical dimension of space (Giessner & Schubert, 2007; Schwartz et al., 1982; Schubert, 2005). In everyday life, there are numerous examples of this association. For instance, we “look up” to people whom we perceive as role models (for instance, people who are committed to doing something good), whereas we “look down” on people, which we do not perceive as equal to ourselves (for instance, criminals). Studies show that when people see drawings with two persons, they judge the more elevated person as more dominant (Schwartz et al., 1982). Further, longer vertical lines in organizational charts increase judged power (Giessner & Schubert, 2007). Those results indicate that the concept of power is partly represented in spatial terms as a vertical difference.

Power can be defined as a capacity to influence other people. This capacity depends on the control of resources (e.g., rewards, information) that are valued or needed by others and that make them dependent on the influence of the person enacting power. Different types of resources result in different types of power (Turner, 2005). For instance, power may be based in legislation, in the ability to reward or punish someone, or it may rely on the availability of information (Raven, 1993). Thus, power is a diverse concept and what power exactly means depends on the context.

For the present study, we developed a new paradigm to study the grounding of abstract concepts. In the past diverse methods have been used such as generating associations (e.g., Harpaintner et al., 2018), priming tasks (e.g., Giessner & Schubert, 2007), compatibility tasks (Schubert. 2005; Zwaan & Yaxley, 2003), or dual-task methods (e.g., Richardson et al., 2003). In the visual-spatial domain pictures have also been used. For instance, participants judge pictures on a certain dimension (Schwartz et al., 1982), participants decide which image of abstract objects relations fits a verbal description best (e.g., Richardson et al., 2003, Exp. 1; Schubert, 2005, Exp. 1), or participants arrange given shapes themselves (e.g., Richardson et al., 2003, Exp. 2). In the present study, we decided to apply a new approach. We combined visual imagery and drawing to provide converging evidence to other studies for the grounding of abstracts concepts. Drawings can be thought of as a visible representation of mental representations and may be used to study aspects of cognition (Fan et al., 2023).

We chose pairs of concepts that denote more or less exertion of power, and that tap into different bases of power. We chose the concept pairs forbiddance/precept and dictatorship/democracy as indicators of legitimate power. We further chose the concept pair wealth/poverty as an indicator of the general availability of resources that may be related to referent power. Further we chose the concepts of experience/naivety, and wisdom/foolishness, which may be related to expert power. Note that the valance of the concepts related to more power differs between concepts pairs. Whereas dictatorship implies the exertion of more power than democracy, democracy has a more positive valence than dictatorship. In the concept pair wealth/poverty, however, the exertion of more power and the more positive valence both coincide in the concept wealth. This was done because positive valence, like more power, is represented higher in space than negative valence (Meier & Robinson, 2004). We wanted to differentiate between effects of valence and effects of power. We presented participants only with one concept at a time, such that no direct comparison between related concepts took place. We asked participants to attend to the image that came to their mind when they read a concept, then asked them a few questions about the image, and finally asked them to draw a sketch of the image in a frame.

We analyzed drawings with respect to their spatial characteristics. We expected that drawings of concepts associated with more power are drawn higher, that is, they have a higher vertical center (cf. Schubert, 2005). Even though an association of power with vertical position has been previously reported, in those studies a direct comparison of two concepts was often made. We were interested to see whether such an effect would still be observable when concepts are presented in isolation. We further expected that the vertical extension of concepts associated with more power might be larger than the vertical extension of concepts associated with less power (Giessner & Schubert, 2007). We had no specific expectation regarding the center and the extension of the drawings on the horizontal dimension.

The questions concerning images were designed to capture several aspects of the images. One set of questions referred to the *imaginability of the concepts.* Those questions were derived from the concept of imagery ability (Collet et al., 2011; Cumming & Eaves, 2018; McAvinue & Robertson, 2007) and concerned*,* for example, whether participants had a spontaneous image or whether they had to think about it. Though imagery ability is regarded as a trait, imagery ability also depends on expertise or familiarity with the imagined content (e.g., Reed 2002), and may further depend on the content itself (e.g., Munzert 2008). Thus, those questions were thought to indicate the visual imaginability of the concepts. We expected it might be easier to create a visual image for some concept pairs (e.g., for wealth/poverty) than for others (e.g., wisdom/foolishness), consistent with literature showing that different abstract concepts are associated with different modalities (Harpaintner et al., 2018).

A further set of questions concerned *qualitative aspects of the image*, for example, whether it was in color or in black and white. In those questions we expected to observe effects of power. For instance, more powerful concepts should result in more colorful images. However, we also expected to observe some differences depending on valence and the specific content of concepts. In particular, democracy, which has a more positive valence and is associated with greater diversity than dictatorship, should result in a more colorful image.

A final set of questions concerned the *content of images*, for example, whether animate beings (people or animals), inanimate objects or abstract content (lines and shapes) were part of the image. Here, we did not have any specific expectations regarding the influence of power, but expected that similar themes would be expressed in high- and low-power concepts of a concept pair. To support this analysis, we further categorized the content of drawings.

## Methods

### Participants

Originally 162 participants took part in the study. Of those, 14 were excluded for the following reasons: (a) drawings could not be analyzed (in at least one condition, the drawing was missing, participants wrote only a word, or the drawing extended the assigned frame; ten participants) and (b) the concept precept was understood in a different way to what was originally meant (the German word “Gebot” can mean either “precept” or “bid”; four participants). The age of the remaining 148 participants was between 18 and 64 years (*M* = 30.6 years, *SD* = 12.4). Fifty-seven identified as male and 91 identified as female. The highest level of education varied: secondary school (*n* = 2), vocational education (*n* = 33), A-level (*n* = 55), Bachelor’s degree (*n* = 35), Master’s degree (*n* = 15), and other (*n* = 8). Participants were recruited from university students and friends and relatives of the experimenters in Austria and Germany. All participants gave informed consent. The study was approved by the local ethics committee. Students received course credit for participating in the study.

To confirm the appropriateness of our sample size, we used G*Power 3.1.9.7 (Faul et al., 2007) to calculate the required sample size for the concept pair and power interaction. We choose F-test (ANOVA, repeated measures, within factors) as the type of test. The number of measurements was set at five and the number of groups was set at one.^1^ Statistical significance was set at *p* < .05. A small effect-size of η_p_^2^ = 0.1 was assumed. The required sample size to achieve a power of 0.9 is a minimum of 37 participants. A larger sample was recruited, however, because we wanted a large sample of drawings to look at their content.

### Materials and procedure

The data were collected using paper and pencil. For the present study, ten concepts grouped into five pairs were used. In each of the concept pairs, one of the concepts was associated with relatively more power or relatively more exertion of power in comparison to the other concept. Concept pairs were: forbiddance/precept, dictatorship/democracy, wealth/poverty, experience/naivety, and wisdom/foolishness. The concepts were chosen to tap into different bases of power (see *Introduction*). Nine raters all agreed on which of the two concepts was associated with relatively more power or relatively more exertion of power in comparison to the other concept.

In a trial, participants were randomly presented with one concept at a time. Upon presentation of the concept, they were asked to think about the concept and to attend to visual images they might have when thinking about it. Then, they were asked several questions concerning the images in their mind, which were answered on 7-point semantic differential or rating scales. Three questions were related to imagery ability (Collet et al., 2011; Cumming & Eaves, 2018; McAvinue & Robertson, 2007): the spontaneity of the images (necessity to contemplate about an image vs. spontaneous image), clearness (blurred vs. clear), and vividness (not vivid vs. very vivid). Three questions concerned the visual quality of the images: color (black/white/grey vs. colorful), color intensity (weak vs. strong), brightness (dark vs. bright). One question concerned movement within the image (static vs. dynamic) and three questions referred to the content of images: animate beings (persons or animals), inanimate objects, and abstract content (not at all – solely).

Participants were then asked to draw a sketch of their image in an 8 cm x 8 cm square using a pencil. They were told that the sketch was not required to be beautiful or detailed, but that it should represent a rough representation of their image. Participants could write a comment on their drawing if they wanted to, to note what the drawing was supposed to mean.

At the start of a session, participants first reported their demographic data, and then three tasks were performed in a counterbalanced order. One of the three tasks included the visual images of power-related concepts, of which the data are reported here. The data from the other two tasks will be reported elsewhere. Note that there were no effects of task position (first, second, or third) that might challenge the present results or interpretations (see Online Supplemental Material (OSM)).

### Data analysis

Data were analyzed using jamovi Version 2.3.38 (The jamovi Project, 2022). To analyze the drawings, the lowest and highest position, as well as the leftmost and rightmost position of the drawing in the frame was measured in each drawing. From those coordinates, we calculated the following variables: vertical extension, vertical center (distance of the center relative to the top line of the square), horizontal extension, and horizontal center (distance of the center relative to the left line of the square).

Those different spatial characteristics of the drawings as well as the different questions concerning the imaginability of concepts and the qualities of images were analyzed using repeated-measures analyses of variance (ANOVA) with the factors concept pair (forbiddance/precept, dictatorship/democracy, wealth/poverty, experience/naivety, wisdom/foolishness) and power (high, low). Questions concerning the content of drawings were analyzed using ANOVAs with the factors content (animate beings, inanimate objects, abstract content) and power (high, low), separately for each concept pair. If Mauchly´s test indicated that the assumption of sphericity was violated, Greenhouse-Geisser-corrected degrees of freedom and p-values are reported. Post hoc significance values were adjusted for multiple testing using Tukey’s correction. When several post hoc comparisons are reported together, minimum (p_min_) or maximum p-values (p_max_) are reported.

We additionally performed an analysis of the content of drawings. For this, we looked at the drawings and decided on coding categories separately for each concept pair. Coding categories differed between concept pairs because we wanted to capture recurrent topics and possible differences between high-power and low-power concepts. For each participant and each drawing, we coded the presence of each category. Note that most of the categories were not mutually exclusive. Thus, the presence of several elements could be coded for a drawing.

## Results

### Spatial characteristics of drawings

**Fig. 1:**
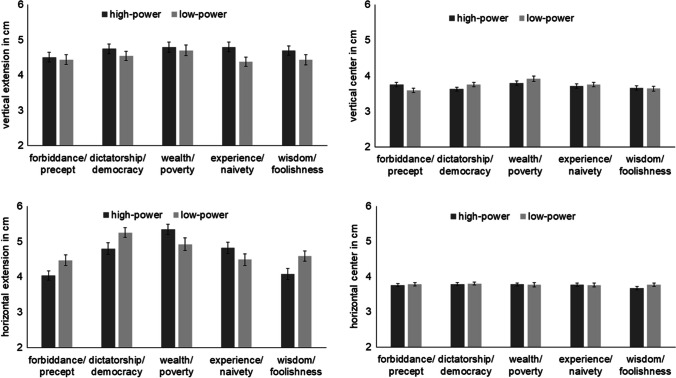
**S**patial characteristics of drawings in an 8 cm x 8 cm square. Vertical extension, vertical center (distance of the center relative to the top line of the square; a lower value means the drawing is higher), horizontal extension, and horizontal center (distance of the center relative to the left line of the square) depending on concept pair (forbiddance/precept, dictatorship/democracy, wealth/poverty, experience/naivety, wisdom/foolishness) and power (high, low)

In Fig. 1, means and standard errors of vertical extension, horizontal extension, vertical center, and horizontal center depending on concept pair (forbiddance/precept, dictatorship/democracy, wealth/poverty, experience/naivety, wisdom/foolishness) and power (high, low) can be seen. The results of the ANOVAs are shown in Table 1. Because the main effect of concept pair was not of theoretical interest in this analysis, we only refer to the main effect of power and the interaction between concept pair and power in the text.

**Table 1:** Spatial characteristics of drawings

	F	df	p	η^2^_p_
*Vertical extension*				
Concept pair	1.563	4, 588	0.183	0.011
Power	11.810	1, 147	< .001	0.074
Concept pair x power	0.816	3.75, 551.57	0.509	0.006
*Vertical center*				
Concept pair	4.964	3.75, 550.68	< .001	0.033
Power	0.478	1, 147	0.491	0.003
Concept pair x power	2.505	3.73, 547.77	0.045	0.017
*Horizontal extension*				
Concept pair	16.34	3.78, 555.58	< .001	0.100
Power	2.52	1, 147	0.115	0.017
Concept pair x power	6.51	3.80, 559.24	< .001	0.042
*Horizontal center*				
Concept pair	0.733	3.72, 547.16	0.561	0.005
Power	0.786	1, 147	0.377	0.005
Concept pair x power	0.521	4, 588	0.720	0.004

Results of the ANOVAs with the factors concept pair (forbiddance/precept, dictatorship/democracy, wealth/poverty, experience/naivety, wisdom/foolishness) and power (high, low) on the spatial characteristics of drawings

#### Vertical extension

The significant main effect of power indicated that the vertical extension was larger in high-power (*M* = 4.71 cm) than in low-power concepts (*M* = 4.50 cm). The interaction between concept pair and power was not significant.

#### Vertical center

The main effect of power was not significant. The interaction between concept pair and power was significant. However, post hoc comparisons did not reveal significant differences between high-power and low-power concepts within any concept pair (*p*_min_ = 0.257).

#### Horizontal extension

The main effect of power was not significant. The interaction between concept pair and power was significant. However, none of the post hoc comparisons between high- and low-power concepts within each concept pair reached significance (*p*_min_ = .109)

#### Horizontal center

The main effect of power and the interaction between concept pair and power were not significant.

### Imaginability of concepts and qualities of images

**Fig. 2:**
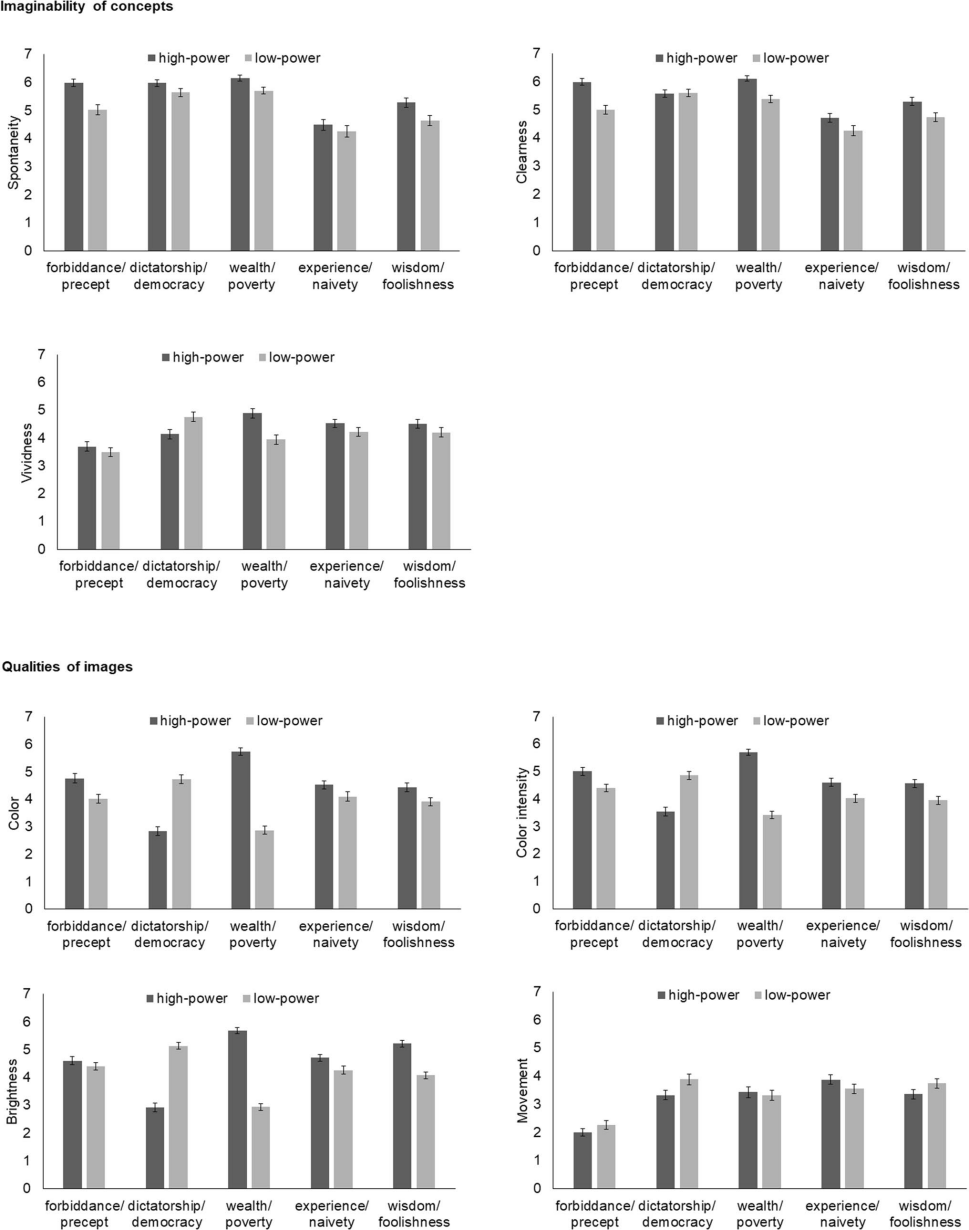
Questions concerning imaginability of concepts and qualities of images. Means and standard errors of the ratings of the images, depending on concept pair (forbiddance/precept, dictatorship/democracy, wealth/poverty, experience/naivety, wisdom/foolishness) and power (high, low)

Means and standard errors of the ratings concerning the imaginability of concepts and qualities of the images are shown in Fig. 2. Results of the ANOVAs can be seen in Table 2. Note that we will describe the main effect of concept pair only for the dependent variables related to the imaginability of concepts (spontaneity, clearness, and vividness), as it was of theoretical interest here to investigate whether the ability to create a visual image differed between concepts pairs. For the other ratings, we only refer to the main effect of power and the interaction between concept pair and power.

**Table 2:** Questions concerning imaginability of concepts and qualities of images

	F	df	p	**η**^**2**^_**p**_
*Spontaneity*				
Concept pair	39.04	3.49, 512.46	< .001	0.210
Power	35.78	1, 147	< .001	0.196
Concept pair x power	2.60	3.38, 497.37	0.045	0.017
*Clearness*				
Concept pair	29.71	3.68, 540.44	< .001	0.168
Power	48.15	1, 147	< .001	0.247
Concept pair x power	5.58	3.69, 543.01	< .001	0.037
*Vividness*				
Concept pair	11.98	3.64, 535.76	< .001	0.075
Power	6.69	1, 147	0.011	0.044
Concept pair x power	8.89	3.72, 546.36	< .001	0.057
*Color*				
Concept pair	7.63	3.75, 551.77	< .001	0.049
Power	44.39	1, 147	< .001	0.232
Concept pair x power	68.38	3.24, 476.66	< .001	0.317
*Color intensity*				
Concept pair	6.57	3.57, 524.49	< .001	0.043
Power	61.92	1, 147	< .001	0.296
Concept pair x power	56.04	3.38, 496.98	< .001	0.276
*Brightness*				
Concept pair	9.05	3.50, 514.50	< .001	0.058
Power	41.74	1, 147	< .001	0.221
Concept pair x power	122.00	3.37, 495.98	< .001	0.454
*Movement*				
Concept pair	35.99	3.62, 531.97	< .001	0.197
Power	2.30	1, 147	0.131	0.015
Concept pair x power	3.00	3.40, 499,69	0.025	0.020

Results of the ANOVAs with the factors concept pair (forbiddance/precept, dictatorship/democracy, wealth/poverty, experience/naivety, wisdom/foolishness) and power (high, low) on the ratings of the images

#### Imaginability of concepts

##### Spontaneity

The main effect of concept pair indicated that visual images were significantly more spontaneous for forbiddance/precept, dictatorship/democracy, and wealth/poverty than for experience/naivety and wisdom/foolishness (*p*_max_ = .003). Further, visual images were significantly more spontaneous for wisdom/foolishness than for experience/naivety (*p* = .004) and visual images were significantly more spontaneous for wealth/poverty than for forbiddance/precept (*p* = .011). The spontaneity of the visual images for dictatorship/democracy did not significantly differ from wealth/poverty (*p* = 0.772) or forbiddance/precept (*p* = 0.134).

The main effect of power was modified by a significant interaction between concept pair and power. High-power concepts were significantly more spontaneously imagined in the concept pairs forbiddance/precept (*p* < .001) and wealth/poverty (*p* = .006). In the other concept pairs, no significant differences were observed (*p*_min_ = .087)

##### Clearness

The main effect of concept pair indicated that visual images were significantly clearer for forbiddance/precept, dictatorship/democracy, and wealth/poverty than for experience/naivety and wisdom/foolishness (*p*_max_ = .003). Visual images were significantly clearer for wisdom/foolishness than for experience/naivety (*p* = .002). All other differences were not significant (*p*_min_ = .215).

The main effect of power was modified by a significant interaction between concept pair and power. High-power concepts were imagined significantly clearer in the concept pairs forbiddance/precept (*p* < .001), wealth/poverty (*p* < .001), and wisdom/foolishness (*p* = .046). In the other concept pairs, no significant differences were observed (*p*_min_ = .258).

##### Vividness

The significant main effect of concept pair indicated that visual images were significantly less vivid for forbiddance/precept than for all other concept pairs (all *p* < .001). All other differences were not significant (*p*_min_ = .976). The significant main effect of power was modified by a significant interaction between concept pair and power. For the concept pair wealth/poverty, the higher power concept was rated as significantly more vivid (*p* <.001). All other comparisons did not reach significance (*p*_min_ = .111).

#### Qualities of images

##### Color

The significant main effect of power was modified by a significant interaction between concept pair and power. Whereas images for more powerful concepts were significantly more colorful than for less powerful concepts for the concept pairs forbiddance/precept (*p* = .033) and wealth/poverty (*p* < .001), the reverse was the case for dictatorship/democracy (*p* < .001). No significant differences were observed in the other two concept pairs (*p*_min_ = .123).

##### Color intensity

The significant main effect of power was modified by the significant interaction between concept pair and power. Color was rated as more intense in the more powerful concept in all concept pairs (*p*_max_ = .035), apart from dictatorship/democracy, in which the reverse was observed (*p* <.001).

##### Brightness

The significant main effect of power was modified by the significant interaction between concept pair and power. Whereas more powerful concepts were imagined brighter in the concept pairs wealth/poverty (*p* <.001) and wisdom/foolishness (*p* < .001), the reverse was the case for dictatorship/democracy (*p* < .001). No significant differences were observed in the other two concept pairs (*p*_min_ = .108).

##### Movement

The main effect of power was not significant. The interaction between concept pair and power was significant, but the post hoc comparisons between the high- and low-power concept were not significant for any of the concept pairs (*p*_min_ = .461).

### Content of images and content of drawings

**Fig. 3:**
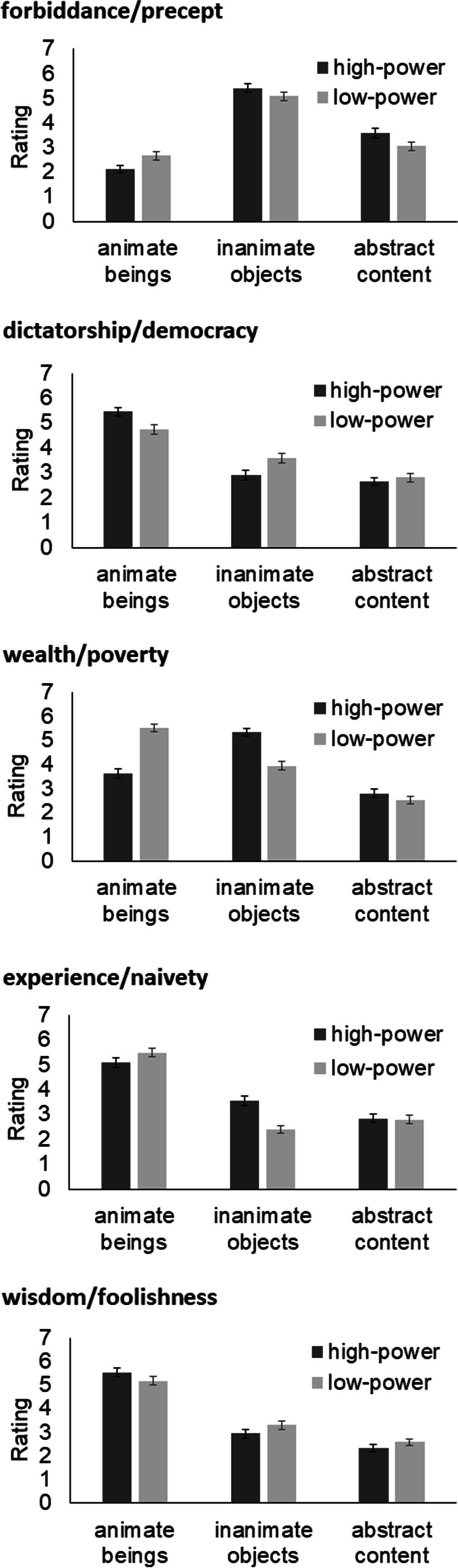
Questions concerning the content of images. Means and standard errors of the ratings of the images, depending on content (animate beings, inanimate objects, abstract content) and power (high, low)

In Fig. 3, means and standard errors of the ratings of the images, depending on content (animate beings, inanimate objects, abstract content) and power (high, low) can be seen, separately for each concept pair. In Table 3, results of the respective ANOVAs are shown. In Table 4, the results of the content analysis of the drawings are shown, i.e., the frequencies of certain elements in the drawings. See OSM for sample drawings of each concept.

**Table 3:** Questions concerning the content of images

	F	df	p	**η**^**2**^_**p**_
Forbiddance/precept				
Power	1.76	1, 147	0.187	0.012
Content	92.43	2, 294	< .001	0.386
Power x content	7.19	1.92, 281.63	0.001	0.047
Dictatorship/democracy				
Power	0.333	1, 147	0.565	0.002
Content	75.335	1.82, 267.56	< .001	0.339
Power x content	7.372	1.68, 247.55	0.002	0.048
Wealth/poverty				
Power	0.961	1, 147	0.328	0.006
Content	66.009	2, 294	0.011	0.310
Power x content	51.778	1.62, 237.97	< .001	0.260
Experience/naivety				
Power	10.3	1, 147	0.002	0.065
Content	94.7	1.81, 265.92	< .001	0.392
Power x content	13.5	1.85, 272.34	< .001	0.084
Wisdom/foolishness				
Power	0.905	1, 147	0.343	0.006
Content	111.626	1.76, 258.55	< .001	0.432
Power x content	3.627	1.84, 269.88	0.031	0.024

Results of the ANOVAs with the factors power (high, low) and content (animate beings, inanimate objects and abstract content) on the ratings of images

**Table 4:** Frequency of elements in drawings

	High-power	Low-power
	Forbiddance	Precept
Traffic signs	85	27
Traffic situations	7	4
Sigs (other than traffic)	22	2
Ten precepts	0	59
Bible or church (other than 10 precepts)	0	28
	Dictatorship	Democracy
Political parties or leaders (past or present)	60	23
One person in a hierarchical relation with others	50	11
Single person	18	6
Several persons with no obvious hierarchy	1	47
Voting (objects or actions)	0	34
	Wealth	Poverty
Money	94	23
Housing/Sleeping	31	29
Valuable Objects	21	3
Friends, Family, Relationship	32	13
Health	4	1
Food	3	22
Depiction of a person	54	112
Actions	4	53
	Experience	Naivety
Number of persons (0 / 1 / ≥ 2)	44 / 78 / 26	42 / 79 / 27
Sex of person (female / male / unclear)	11 / 37 / 33	37 / 3 / 39
Age of person (old / child to medium age / unclear)	35 /5 / 41	0 / 43 / 40
Events or actions	22	23
	Wisdom	Foolishness
Number of persons (0 / 1 / ≥ 2)	49 / 90 / 9	50 / 83 / 15
Sex of persons (female / male / unclear)	4 / 69 /28	5 /12 / 68
Age of person (old / child to medium age / unclear)	36 / 0 / 56	0 / 8 / 77
Events or actions	11	31

In most cases, elements are not mutually exclusive, i.e., for one participant (N = 148) several elements could be coded

#### Forbiddance and precept

In the analysis of the ratings of the images, the significant main effect of content indicated that the presence of inanimate objects was rated higher than the presence of animate beings and abstract content, and that the presence of abstract content was rated higher than the presence of animate beings (all *p* <.001). The significant interaction between power and content indicated that the presence of abstract content was rated higher in forbiddance than in precept (*p* = .030), but no significant differences were observed in animate beings and inanimate objects (*p*_min_ = .069).

The analysis of the content of drawings was consistent with the ratings of images. Drawings for forbiddance very often contained signs, mostly related to road traffic. Other signs, for instance related to smoking or drugs, were depicted as well. Sometimes traffic lights or more complex situations were depicted. Signs were also sometimes modified compared to the original to put emphasis on the aspect of forbiddance. Drawings for precept sometimes consisted of traffic signs as well. More often, however, a reference was made to religion, notably the ten precepts. Other church or bible related depictions were observed as well.

#### Dictatorship and democracy

In the analysis of the ratings of images, the significant main effect of content indicated that the presence of animate beings was rated higher than the presence of inanimate objects and abstract content (both *p* <.001), and that the presence of inanimate objects was rated higher than the presence of abstract content. The significant interaction between power and content approached significance and indicated that the presence of animate beings was rated higher in dictatorship than in democracy (*p* = .050), whereas the presence of inanimate objects was rated higher in democracy than in dictatorship (*p* = .029). No significant difference between dictatorship and democracy was observed in the ratings of abstract content (*p* = .971).

The analysis of drawings showed that for both concepts – though more often for dictatorship – parties, countries, or political leaders (past or present) were depicted to indicate the political system. For democracy, a lot of drawings referred to voting of political parties, either by showing a ballot paper or a person putting a ballot paper into a ballot box. Frequently, drawings contained persons. In dictatorship, those persons were often depicted in a hierarchical relation to each other, whereas in democracy, often several persons without obvious differences in hierarchy were depicted, emphasizing all people as a group. Some of the drawings for democracy also emphasized diversity, for example, differences in opinion between people, or depicted political demonstrations.

#### Wealth and poverty

In the ratings of images, the significant main effect of content indicated that the presence of animate beings and inanimate objects was rated higher than the presence of abstract content (both *p* < .001). The ratings for animate beings and inanimate objects did not significantly differ from each other (*p* = .965). The significant interaction between power and content indicated that the presence of animate beings was rated higher in poverty than in wealth (*p* < .001), whereas the presence of inanimate objects was rated higher in wealth than in poverty (*p* < .001).

The analysis of drawings showed that money was a recurrent theme in wealth. In nearly two-thirds of participants at least one part of the drawing was devoted to that topic. Direct depictions of money were also observed in poverty (here a lack of money was indicated). Participants further drew houses or sleeping environments (e.g., under a bridge for the concept of poverty). For wealth, status symbols such as a boat or a (presumably) expensive necklace were drawn. Friends, family, and relationships to other people were further indicators for wealth, whereas the lack of such was indicated in drawings of poverty. Food was more often drawn for poverty than for wealth. In poverty, more depictions of persons and actions were observed; actions often consisted of a person begging.

#### Experience and naivety

In the analysis of the ratings of images, the significant main effect of content indicated that the presence of animate beings was rated higher than the presence of inanimate objects or abstract content (both *p* <.001); the latter two did not significantly differ between each other (*p* = .623). The significant main effect of power was modified by the significant interaction between power and content. The presence of inanimate objects was rated higher in experience than in naivety (*p* < .001); the other comparisons between experience and naivety did not reach significance (*p*_min_ = .432)

Because a lot of drawings contained persons, we focused on the characteristics of those persons in the analysis of drawings. Though often no obvious cues to the sex of a person were present in the drawings, those cases in which cues (like a beard or long hair) were present indicate that males were drawn more often for experience, whereas females were drawn more often for naivety. Similarly, when a cue to the age of a person was present in the drawings, older age was depicted more often in the drawings of experience, whereas children, younger people, or people of medium age were depicted more often in drawings for naivety.

#### Wisdom/foolishness

In the analysis of the ratings of images, the significant main effect of content indicated that the presence of animate beings was rated higher than the presence of inanimate objects and abstract content, and that the presence of inanimate objects was rated higher than the presence of abstract content (all *p* < .001). The interaction between power and content was significant, but none of the post hoc comparisons between wisdom and foolishness reached significance (*p*_min_ = .338).

The analysis of drawings again focused on the characteristics of persons in the drawings, because again a lot of drawings contained persons. Drawings indicated that wisdom was associated with older age and male sex. Foolishness was not systematically associated with sex or age. One recurring theme in foolishness was drugs (either in the form of objects or as the action of taking drugs). More events or actions were drawn for foolishness than for wisdom. Example actions from the drawings were drunk driving or jumping off a cliff.

## Discussion

In the present study, we developed a new paradigm to study the grounding of abstract concepts by using a combination of visual imagery and drawing. We investigated the grounding of abstract concepts related to more or less (exertion of) power. Upon presentation with a concept, participants were asked to create a visual image. They then answered questions concerning the visual image regarding imaginability, qualities, and content. Afterwards they drew a sketch of it. Results showed that drawings of more powerful concepts had a larger vertical extension than drawings of less powerful concepts. Some results regarding imaginability and quality of the visual images depended on the specific content of the concepts. For instance, sometimes images of more powerful concepts were rated as more colorful (e.g., wealth vs. poverty) and sometimes as less colorful (e.g., dictatorship vs. democracy). Images and drawings often contained persons or objects but were rarely abstract.

### Spatial characteristics of drawings

Contrary to our expectations, the vertical center of high-power concepts was not higher than the vertical center of low-power concepts. This is in contrast to other studies that observed an association between the vertical position and power (e.g., Schubert, 2005). The diverging results may be explained by differences in methodology. In previous studies, stimuli of high- and low-power concepts were often presented at the same time on different positions of the screen, which may have emphasized the vertical position, whereas in the present study, only one concept was presented at a time. In future studies it might be interesting to present concepts at the same time and to have participants draw a picture containing both.

However, consistent with our expectations, high-power concepts showed a larger vertical extension than low-power concepts. This is consistent with the observation that longer vertical lines in organizational charts increase judged power (Giessner & Schubert, 2007). Thus, even though vertical position was not influenced by power differences, vertical extension was. We consider this strong evidence for the assumption that power is related to the vertical dimension, as this effect occurred even though concepts were presented randomly one at a time.

This is further corroborated by the analysis of the content of drawings. In drawings for dictatorship, often one versus several other persons were depicted in a hierarchical relation to each other. In democracy, when people were depicted, they often were drawn on the same level.

As expected, no systematic effects were observed on the horizontal dimension, neither in the horizontal center, nor in the horizontal extension. Thus, the horizontal dimension is not related to power.

### Visual imaginability of concepts

Visual images were less spontaneous and less clear for experience/naivety and wisdom/foolishness than for the other concepts. Consistent with previous studies, this indicates that abstract concepts differ in their grounding in the visual modality (Harpaintner et al., 2018, 2022). Alternatively, this effect may not only be due differences in grounding in the visual modality, but to differences in grounding per se. One might speculate that in everyday life people have more experience with situations related to forbiddance/precept, wealth/poverty, and dictatorship/democracy compared to experience/naivety and wisdom/foolishness. People may be frequently confronted with the concepts of forbiddance and precept when they navigate through traffic or with respect to the rules in society. Dictatorship and democracy are concepts that are frequently encountered in the media. Likewise, wealth and poverty are frequent concepts in the media and people may often see beggars on the street. Actual experiences with the concepts experience/naivety and wisdom/foolishness may be less frequent.

Interestingly, in some high-power concepts visual images were more spontaneous (forbiddance/precept, wealth/poverty) and clearer (forbiddance/precept, wealth/poverty, and wisdom/foolishness) than low-power concepts. This is similar to an effect found previously that reaction times are faster when participants have to find the powerful than when they have to find the less powerful group (Schubert, 2005). Schubert (2005) provided several possible explanations for this effect, some of which may also apply to the present study. One explanation is that powerful concepts may be more important than powerless concepts, that they therefore receive more attention, and that symbolic representations are therefore clearer (Schubert, 2005). Another explanation is that concepts related to more power are “easier” and that concepts related to less power involve more elaborate thinking. This might be due to less familiarity with thinking about less power or that the power dimension is asymmetric (Schubert, 2005). Note that the observation is also consistent with judgement preference effects. For instance, it is easier to judge whether a tone is louder than to judge whether it is softer (Intons-Peterson, 1980), and it is easier to find a larger than a smaller number (Parkman, 1971).

Apart from spontaneity and clearness, we also asked participants about the vividness of their visual images to investigate the imaginability of concepts. However, when we look at the data patterns, it seems that vividness ratings resemble more the data from the variables we used to investigate the qualities of the images.

### Qualities of visual images

Color, color intensity, and brightness showed similar patterns. In most concept pairs (except for dictatorship/democracy), the higher power concept was rated as being more colorful (significant for forbiddance/precept and wealth/poverty), as having a higher color intensity, and as being brighter (significant for wealth/poverty and wisdom/foolishness). We think that the observed effects may at least partly be attributed to power differences. As outlined above, powerful concepts may receive more attention or may be “easier,” thereby resulting in clearer symbolic representations (Schubert, 2005). This may in turn not only affect spontaneity and clearness of visual images but may also result in more colorful, color intense, and brighter images.

The particularly strong and consistent effects for the concept pair wealth/poverty (wealth was also imagined more vividly than poverty) may be additionally explained by the more positive valence of wealth compared to poverty. More brightness is associated with a more positive valence (Meier et al., 2015). Further, achromatic colors such grey and black are associated with more negative emotions, whereas chromatic colors are for the most part associated with more positive emotions (Jonauskaite et al., 2020). Similarly, for wisdom/foolishness (only color intensity and brightness were significant) and experience/naivety (only color intensity was significant) power has a more positive valence. However, the effects are somewhat weaker and less consistent than for the concept pair wealth/poverty. We think this can be explained by the content of images. Naivety was in some instances associated with younger age and female gender (see below), which may in turn be associated with being more colorful and brighter than older age (with which both experience and wisdom were associated).

Most markedly, in the concept pair dictatorship/democracy the opposite pattern was observed: the low-power concept was imagined to be more colorful, with more color intensity and brighter than the high-power concept. Again, this effect may be explained by the valence of the concepts, which is more positive for democracy than for dictatorship, resulting in more brightness and more chromatic colors (Jonauskaite et al., 2020; Meier et al., 2015). Further, democracy is associated with more diversity, and may thus, metaphorically, be more colorful.

Movement in images did not significantly differ between high- and low-power concepts. Whether movement occurred in the images may depend on the specific content of the images and images may be more dynamic when actions and situations were chosen to depict the situation (e.g., someone giving money to someone else who is begging for the concept of poverty) than when symbols were used (e.g., money that is crossed out for poverty).

### Power and valence

The present results indicate that power is associated with vertical extension and with more color, more color intensity, and more brightness. As mentioned above, valence, in addition to power, may have partly contributed to the results. Like more power, positive valence is represented higher in space than negative valence (Meier & Robinson, 2004) and it may therefore also be similar to power in other aspects. Thus, one may raise the question if the results cannot be explained solely by differences in valence. Schubert (2005) argued that valence and power may often coexist in real life. We had one concept pair in which this was not the case (dictatorship/democracy) for which partly different results were obtained compared with the other concept pairs.

However, we think that power and valence may both have made unique contributions to the present results. First, qualities of the images differed between forbiddance and precept, which are not obviously associated with differences in valence. Second, and more importantly, vertical extension was larger for high-power than for low-power concepts, even in dictatorship/democracy, indicating that power and not valence resulted in this effect.

### Grounding of abstract concepts

Grounded cognition accounts differ in the way they conceptualize grounding of abstract concepts. Lakoff and Johnson (1980a,b) claim that the use of metaphors is the main mechanism through which we comprehend abstract concepts. In the present study, we sometimes observed grounding via verbal metaphors. For instance, one participant drew a bread to depict the concept of foolishness, which probably relates to the verbal expression “dumb like bread.” Another drew a spoon to depict wisdom, which probably relates to the German expression “eating wisdom with a spoon,” meaning that someone thinks they know everything. Likewise, depicting young girls or women for the concepts of naivety and foolishness may also relate to the verbal expression “blonde and dumb” (which is something one participant wrote at the side of the drawing to explain it).

The observation that in experience/naivety and to some degree in wisdom/foolishness stereotypical depictions (old, male vs. young, female) were sometimes drawn to depict the concepts was actually a surprise to us, as equality between sexes is a high value in Austrian and German societies where participants were recruited. The results indicated that those two concept pairs were less likely to evoke spontaneous images than the other concept pairs. Participants therefore were more likely to actively think about a possible image, and it seems particularly shocking that such traditional outdated images result from active thought. However, participants probably did not devote too much time to thinking due to the experimental situation. This may be a situation when old conceptions, conveyed via traditional movies and literature, easily to come to mind.

Other grounded cognition accounts argue that grounding may mainly be based on situations in which concepts are experienced (situated action account; Barsalou, 2008). Direct experience of situations involving abstract concepts rather than metaphors may be central to ground abstract concepts in multimodal simulations (Barsalou & Wiener-Hastings, 2005; Harpaintner et al., 2018). Indeed, the drawings in the present study indicated that experiences, actions, and situations were often the content of the images. For instance, people in Austria and Germany live in democracies in which they can vote. Voting, which is an act of participation in a democracy, was often depicted as a topic in the drawings for democracy. Though most participants most likely did not have a direct experience with dictatorship, they have an indirect experience via frequent confrontation in the news and the media. Other drawings depicted situations and actions like going on vacation (for the concept experience), doing something dangerous (for the concept of naivety), or begging (for the concept poverty), again showing that abstract concepts may relate to specific (direct or indirect) experiences.

Our results are consistent with the observation that the semantic content of grounding can be very diverse and can relate to different modal systems (Harpaintner et al., 2018). Though we used the visual modality to depict the concepts, the content of drawings often implied other modal systems. The content of drawings ranged from objects (traffic signs to denote forbiddance), (social) situations and actions (transfer of knowledge to depict wisdom), (inferred) introspective content (a light bulb for knowing something from experience, being puzzled denoted by question marks for naivety), to emotional content (a heart for indicating love to depict naivety).

Many drawings referred to concrete objects, for example, traffic signs, or money. It is difficult to decide whether those depictions are in favor of the grounding via metaphors or via situated actions. These are all things people have experience with, which may be in favor of the situated action account (Barsalou, 2008, Barsalou & Wiener-Hastings, 2005). However, one may also argue that those objects represent societal metaphors for the abstract concepts (Lakoff & Johnson, 1980a). Interestingly, one and the same object was partly used for different content: A heart was used to depict love as part of the concept of wealth, but also to describe being foolishly in love to depict the concept of naivety. Abstract drawings, depicting lines and shapes that could not directly be associated with a certain meaning, were rarely drawn. Such abstract representations may not be the first thing that comes to mind.

Interestingly, even though some of the concept pairs we used can be regarded as opposites in language, the opposing concepts were not grounded in the same way. This became particularly apparent in wealth/poverty. The presence of animate beings was rated higher in images of poverty than in images of wealth, whereas the reverse was the case for the presence of inanimate objects. This was corroborated by the analysis of the content of drawings. Even though there was some overlap in elements (e.g., money vs. the lack of money), the frequency of this content varied. In particular, in poverty, drawings more often contained persons and actions than in wealth. Thus, opposites in language are not necessarily completely grounded on the same dimensions (see Norman et al., 2011, for a similar discussion on opposites in emotion research).

### Limitations and perspectives

One limitation of the present study is, that it does not definitely rule out alternative accounts arguing that abstract concepts are represented in amodal systems (Mahon & Caramazza, 2008; Mahoon, 2015; Paivio, 1986; Pylyshyn, 1984). However, we think that such accounts would have difficulties with the diversity and richness of content depicted in the drawings and with the observation that different modal systems were implied in the drawings (see Harpaintner et al., 2018, for similar arguments).

One further limitation of the present study is that by asking participants to create visual images and to draw a sketch of those images, we mainly investigated visual representations of abstract concepts. As outlined above, visual images were less spontaneous and less clear for experience/naivety and wisdom/foolishness than for the other concepts, which may indicate that the visual modality may not be very dominant in the representation of those concepts. However, we think most paradigms, at least to some degree, force participants into one modality or another. If visual stimuli are presented on a screen, often visual-spatial representations of abstract concepts are investigated (e.g., Schubert, 2005). If participants provide verbal associations to an abstract concept (e.g., Harpaintner et al., 2018), only representations that can be verbally described can be investigated. Still, our study shows (as do studies using verbal associations, e.g., Harpaintner et al., 2018), that even though one modality is used for the investigation of abstract concepts, conclusions about their grounding in different modalities can be drawn.

In future studies, valence and power may be dissociated more systematically than in the present study. Further, participants may be presented with two concepts differing in power at the same time, and they may be asked to create drawings containing both concepts. Though we think it is important that we were able to show effects related to power differences when concepts are presented in isolation, some effects might be stronger or only become apparent when concepts are presented as a contrast (for instance, an effect on vertical center). Further, in future studies, participants may be asked to create drawings in color or rate colors for their suitability to depict different abstract concepts. It has been shown that some consistent associations between colors and emotions exist (Jonauskaite et al., 2020). This might be the case for other abstract concepts as well.

## Conclusion

In conclusion, we developed a new paradigm, using visual imagery and drawings, which is suitable to investigate the grounding of abstract concepts. We showed that drawings of high-power concepts had a larger vertical extension than drawings of low-power concepts. Qualities of the images depended on the specific concepts. For instance, wealth (higher power) was rated as more colorful than poverty (lower power), but democracy (lower exercise of power) was rated as more colorful than dictatorship (higher exercise of power). These results may partly be explained by the valence of the concepts. Ratings of the content of images and drawings showed that often persons, objects, and situations, but rarely abstract content, were used for grounding. Sometimes drawings contained metaphorical content and sometimes the content of drawings related to specific experiences. Thus, abstract concepts related to power can be depicted visually via grounding in different ways, such as using metaphors, experiences, and actions.

## Supplementary information

ESM 1 (DOCX 2066 kb)

## Data Availability

The data for the experiment are available at https://osf.io/tmc4v/. The experiment was not preregistered.

## Footnotes

1 Note that the interaction between concept pair and power can be equally expressed as a unifactorial ANOVA for repeated measures with concept pair as independent variable and the difference corresponding to the main effect of power (high – low) as dependent variable. Accordingly, we used sample size estimation for unifactorial repeated-measures ANOVA in G*Power.
