# Supplementary material for: Grounding of abstract concepts related to power
Source: Mem Cognit. 2023 Dec 11;53(1):135–49. doi: 10.3758/s13421-023-01492-6 (PMC11779783; doi:10.3758/s13421-023-01492-6)

**SUPPLEMENTAL MATERIAL**

**Grounding of abstract concepts related to power**

Martina Rieger & Victoria K. E. Bart

UMIT TIROL – Private University for Health Sciences and Health Technology, Institute of Psychology, Hall in Tyrol, Austria

**Corresponding author**

Martina Rieger

martina.rieger@umit-tirol.at

**Overview**

This document includes additional content that may be of interest to some readers. First, as the data presented in this paper stem from one of three tasks which were presented in counterbalanced order, one may wonder whether there were any order effects. We therefore analyzed the effects of the position in which the present task was performed (1st, 2nd, or 3rd) on our dependent variables. Note that there were no effects of task position (1^st^, 2^nd^, or 3^rd^) that might challenge the present results or interpretations. Second, we provide sample drawings (including a short description of the content of drawings) to support the content analysis presented in the manuscript.

**Task position**

As the data presented in this paper stem from one of three tasks, which were presented in counterbalanced order, one may wonder whether there were any order effects. We therefore analyzed the effects of the position in which the present task was performed (1st, 2nd, or 3rd) on our dependent variables.

**Spatial characteristics of drawings**

In Table SM1 the results of the ANOVAs with the factors position (1^st^, 2^nd^, 3rd), concept pair (forbiddance/precept, dictatorship/democracy, wealth/poverty, experience/naivety, wisdom/foolishness) and power (high, low) on spatial characteristics of drawings are shown. Note that only the effects involving the factor position are presented.

***Vertical extension:*** None of the effects was significant.

***Vertical center:*** A significant interaction between position and concept pair was observed. However, within each concept pair none of the comparisons between the positions became significant (*p_min_*= .141).

***Horizontal extension:*** None of the effects was significant.

***Horizontal center:*** A significant main effect of position indicated that in the 3^rd^ position, the horizontal center was more rightwards than in the 2nd position (*p* = .029). the interaction between position and power was also significant. However, none of the comparisons between high power and low power for each position separately became significant (*p_min_* = 0.132).

**Table SM1**

*Spatial characteristics of drawings.* *Results of the ANOVAs with the factors position (1^st^, 2^nd^, 3rd), concept pair (forbiddance/precept, dictatorship/democracy, wealth/poverty, experience/naivety, wisdom/foolishness) and power (high, low). Note that only the effects involving the factor position are shown.*

|  | F | df | p | **η²_p_** |
| --- | --- | --- | --- | --- |
| ***Vertical extension*** | | | | |
| Position | 0.709 | 2, 145 | 0.494 | 0.010 |
| Position x concept pair | 1.765 | 8, 580 | 0.081 | 0.024 |
| Position x power | 1.418 | 2, 145 | 0.245 | 0.019 |
| Position x concept pair x power | 0.782 | 8, 580 | 0.619 | 0.011 |
| ***Vertical center*** | | | | |
| Position | 1.16 | 2, 145 | 0.316 | 0.016 |
| Position x concept pair | 2.633 | 8, 580 | 0.008 | 0.035 |
| Position x power | 2.556 | 2, 145 | 0.081 | 0.034 |
| Position x concept pair x power | 0.516 | 8, 580 | 0.845 | 0.007 |
| ***Horizontal extension*** | | | | |
| Position | 0.867 | 2, 145 | 0.423 | 0.012 |
| Position x concept pair | 1.211 | 8, 580 | 0.290 | 0.016 |
| Position x power | 0.444 | 2, 145 | 0.642 | 0.006 |
| Position x concept pair x power | 0.783 | 8, 580 | 0.618 | 0.011 |
| ***Horizontal center*** | | | | |
| Position | 3.37 | 2, 145 | 0.037 | 0.044 |
| Position x concept pair | 1.197 | 8, 580 | 0.298 | 0.016 |
| Position x power | 3.280 | 2, 145 | 0.040 | 0.043 |
| Position x concept pair x power | 1.395 | 8, 580 | 0.195 | 0.019 |

**Imaginability of concepts and qualities of images**

In Table SM2 the results of the ANOVAs with the factors position (1^st^, 2^nd^, 3rd), concept pair (forbiddance/precept, dictatorship/democracy, wealth/poverty, experience/naivety, wisdom/foolishness) and power (high, low) on the ratings of images concerning imaginability of concepts and qualities of images are shown. Note that only the effects involving the factor position are shown.

***Spontaneity:*** None of the effects was significant.

***Clearness:*** None of the effects was significant.

***Vividness:*** The interaction between position, concept pair, and power was significant. This interaction indicated that difference between high-and low-power concepts in wealth/poverty (higher vividness ratings for wealth than for poverty) became more pronounced when the task was performed later.

***Color:*** None of the effects was significant.

***Color intensity:*** None of the effects was significant.

***Brightness:*** None of the effects was significant.

***Movement:*** None of the effects was significant.

**Table SM2**

*Questions concerning imaginability of concepts and qualities of images. Results of the ANOVAs with the factors position (1^st^, 2^nd^, 3^rd^), concept pair (forbiddance/precept, dictatorship/democracy, wealth/poverty, experience/naivety, wisdom/foolishness) and power (high, low) on the ratings of the images. Note that only effects involving the factor position are shown.*

|  | F | df | p | **η²_p_** |
| --- | --- | --- | --- | --- |
| ***Spontaneity*** | | | | |
| Position | 0.539 | 2, 145 | 0.584 | 0.007 |
| Position x concept pair | 0.883 | 8, 580 | 0.530 | 0.012 |
| Position x power | 2.195 | 2, 145 | 0.115 | 0.029 |
| Position x concept pair x power | 0.915 | 8, 589 | 0.504 | 0.012 |
| ***Clearness*** | | | | |
| Position | 0.112 | 2, 145 | 0.894 | 0.002 |
| Position x concept pair | 0.980 | 8, 580 | 0.451 | 0.013 |
| Position x power | 1.213 | 2, 145 | 0.300 | 0.016 |
| Position x concept pair x power | 1.023 | 8, 580 | 0.417 | 0.014 |
| ***Vividness*** |  |  |  |  |
| Position | 0.462 | 2, 145 | 0.631 | 0.006 |
| Position x concept pair | 0.367 | 8, 580 | 0.938 | 0.005 |
| Position x power | 1.474 | 2, 145 | 0.233 | 0.020 |
| Position x concept pair x power | 2.204 | 8, 580 | 0.026 | 0.030 |
| ***Color*** |  |  |  |  |
| Position | 1.020 | 2, 145 | 0.363 | 0.014 |
| Position x concept pair | 0.490 | 8, 580 | 0.864 | 0.007 |
| Position x power | 0.200 | 2, 145 | 0.819 | 0.003 |
| Position x concept pair x power | 1.192 | 8, 580 | 0.301 | 0.016 |
| ***Color intensity*** |  |  |  |  |
| Position | 0.717 | 2, 145 | 0.490 | 0.010 |
| Position x concept pair | 0.6672 | 8, 580 | 0.721 | 0.009 |
| Position x power | 0.0582 | 2, 145 | 0.943 | 0.001 |
| Position x concept pair x power | 0.6319 | 8, 580 | 0.751 | 0.009 |
| ***Brightness*** |  |  |  |  |
| Position | 0.175 | 2, 145 | 0.839 | 0.002 |
| Position x concept pair | 0.307 | 8, 580 | 0.963 | 0.004 |
| Position x power | 0.350 | 2, 145 | 0.705 | 0.005 |
| Position x concept pair x power | 1.407 | 8, 580 | 0.190 | 0.019 |
| ***Movement*** |  |  |  |  |
| Position | 0.0644 | 2, 145 | 0.938 | 0.001 |
| Position x concept pair | 0.707 | 8, 580 | 0.685 | 0.010 |
| Position x power | 1.522 | 2, 145 | 0.222 | 0.021 |
| Position x concept pair x power | 0.724 | 8, 580 | 0.670 | 0.010 |

**Content of images**

In Table SM3 Results of the ANOVAs with the factors position (1^st^, 2^nd^, 3^rd^), power (high, low) and content (animate beings, inanimate objects, abstract content) on the ratings of images can be seen. Only the effects involving the factor position are depicted.

***Forbiddance/precept:*** None of the effects was significant.

***Dictatorship/democracy:*** None of the effects was significant.

***Wealth/poverty:*** None of the effects was significant.

***Experience/naivety:*** None of the effects was significant.

***Wisdom/Foolishness:*** None of the effects was significant.

**Table SM3**

*Content of images. Results of the ANOVAs with the factors position (1^st^, 2^nd^, 3^rd^), power (high, low) and content (animate beings, inanimate objects, abstract content) on the ratings of images concerning the content. Only the effects involving the factor position are presented.*

|  | F | df | p | **η²_p_** |
| --- | --- | --- | --- | --- |
| ***Forbiddance/precept*** | | | | |
| Position | 0.992 | 2, 145 | 0.373 | 0.014 |
| Position x power | 0.0326 | 2, 145 | 0.968 | 0.000 |
| Position x content | 0.3235 | 4, 290 | 0.862 | 0.004 |
| Position x power x content | 0.7004 | 4, 290 | 0.592 | 0.010 |

| ***Dictatorship/democracy*** | | | | |
| --- | --- | --- | --- | --- |
| Position | 1.01 | 2, 145 | 0.367 | 0.014 |
| Position x power | 2.162 | 2, 145 | 0.119 | 0.029 |
| Position x content | 0.853 | 4, 290 | 0.493 | 0.012 |
| Position x power x content | 1.723 | 4, 290 | 0.145 | 0.023 |
| ***Wealth/poverty*** | | | | |
| Position | 0.868 | 2, 145 | 0.422 | 0.012 |
| Position x power | 1.384 | 2, 145 | 0.254 | 0.019 |
| Position x content | 0.122 | 4, 290 | 0.975 | 0.002 |
| Position x power x content | 1.782 | 4, 290 | 0.133 | 0.024 |

| ***Experience/naivety*** | | | | |
| --- | --- | --- | --- | --- |
| Position | 0.775 | 2, 145 | 0.463 | 0.011 |
| Position x power | 0.613 | 2, 145 | 0.543 | 0.008 |
| Position x content | 1.139 | 4, 290 | 0.338 | 0.015 |
| Position x power x content | 0.405 | 4, 290 | 0.805 | 0.006 |
| ***Wisdom/foolishness*** | | | | |
| Position | 1.40 | 2, 145 | 0.251 | 0.019 |
| Position x power | 0.874 | 2, 145 | 0.420 | 0.012 |
| Position x content | 1.362 | 4, 290 | 0.247 | 0.018 |
| Position x power x content | 0.746 | 4, 290 | 0.561 | 0.010 |

**Sample Drawings and Short Description of the Content of Drawings**

**Forbiddance/Precept**

In Figure SM1 samples of participants’ drawings for the concept pair forbiddance/precept are depicted. Drawings for forbidden often contained signs, mostly related to road traffic, but other signs, for instance related to smoking or drugs, were depicted as well. Sometimes traffic lights or more complex situations were depicted. Signs were also sometimes modified compared to the original to put emphasis on the aspect of forbiddance. Drawings for precept were sometimes also traffic signs, but more often references to the bible and the 10 precepts were depicted.

**Figure SM1**

*Samples of Participants’ Drawings for the Concept Pair Forbiddance/Precept*


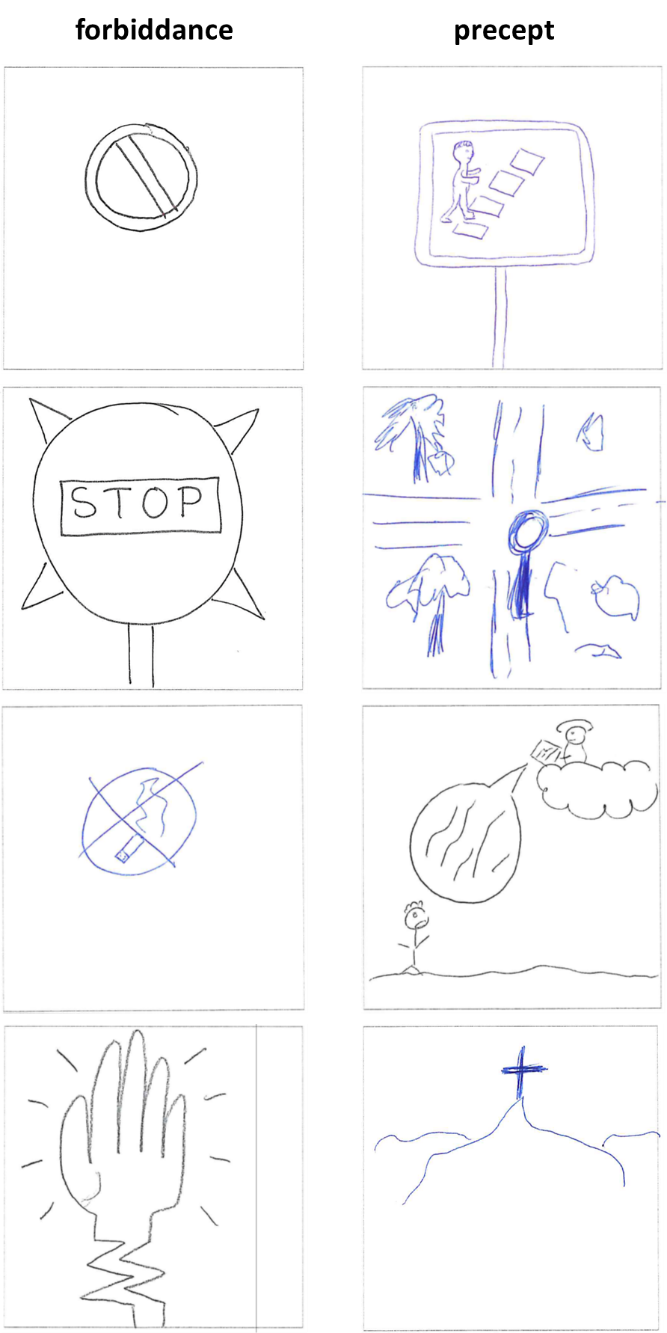


**Dictatorship/Democracy**

In Figure SM2 samples of participants’ drawings for the concept pair dictatorship/democracy are depicted. For both concepts, often parties, countries, or political leaders (past or present) were depicted to indicate the political system, in particular for dictatorship. For democracy, a lot of drawings referred voting of political parties, either by showing a ballot paper or a person putting a ballot paper into a ballot box. Frequently, drawings contained persons. In dictatorship, those persons were often depicted in a hierarchical relation to each other, whereas in democracy, often several persons without obvious differences in hierarchy were depicted, emphasizing all people as a group. Some of the drawings for democracy also emphasized diversity, e.g., differences in opinion, between people.

**Figure SM2**

*Samples of Participants’ Drawings for the Concept Pair Dictatorship/Democracy*


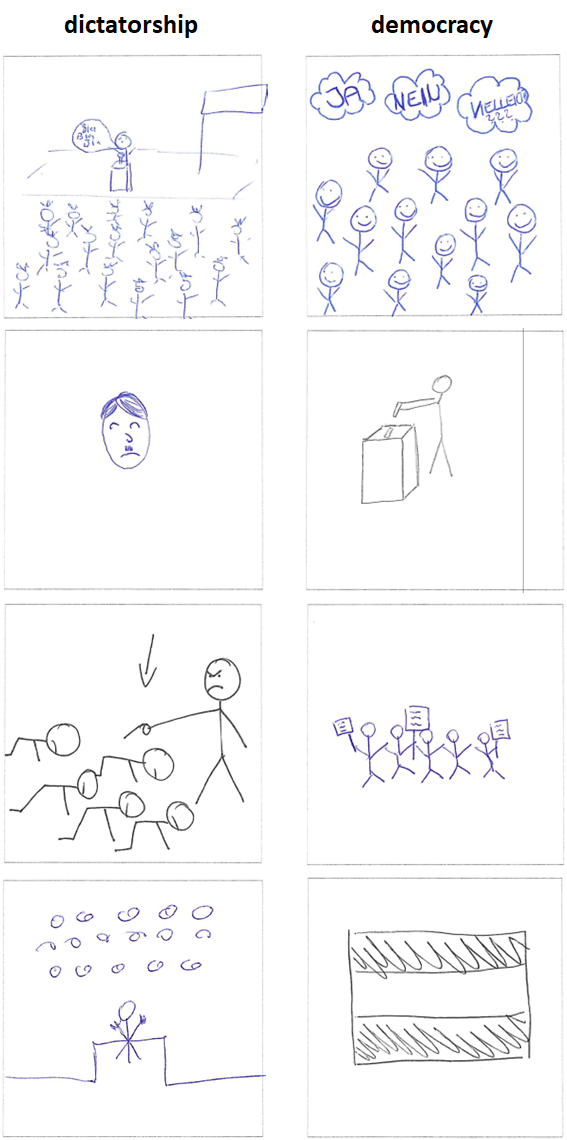


**Wealth/Poverty**

In Figure SM3 samples of participants’ drawings for the concept pair wealth/poverty are depicted. Wealth was often depicted as money. Sometimes status symbols such as a boat and a house were also drawn. However, drawings depicted friends and family as a symbol for wealth as well. In the drawings for poverty, poverty was sometimes depicted as the lack of goods (e.g., money crossed out). More often one central person was depicted, and this person had some attribute for being poor, e.g., they had no home, were begging and/or lonely.

**Figure S3**

*Samples of Participants’ Drawings for the Concept Pair Wealth/Poverty*

*
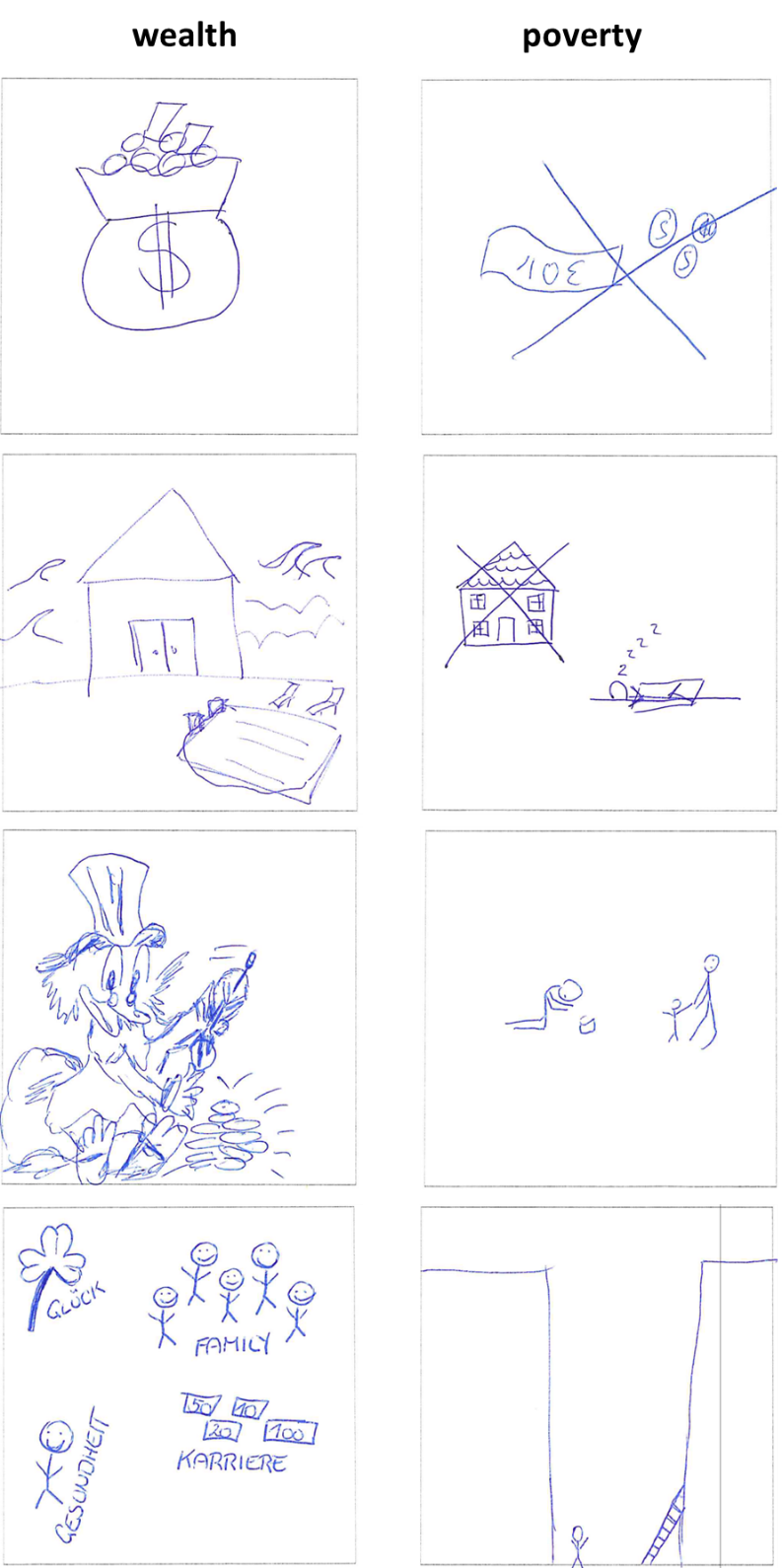
*

**Experience/Naivety**

In Figure SM4 samples of participants’ drawings for the concept pair experience/naivety are depicted. Inanimate objects in drawings for experience contained things like a curriculum vitae. Travelling was sometimes depicted as an action to gain experience. In both concepts persons were often depicted in the drawings. Experience was associated with older age and the male sex, whereas naivety was associated with younger age and the female sex.

**Figure S4**

*Samples of Participants’ Drawings for the Concept Pair Experience/Naivety*

*
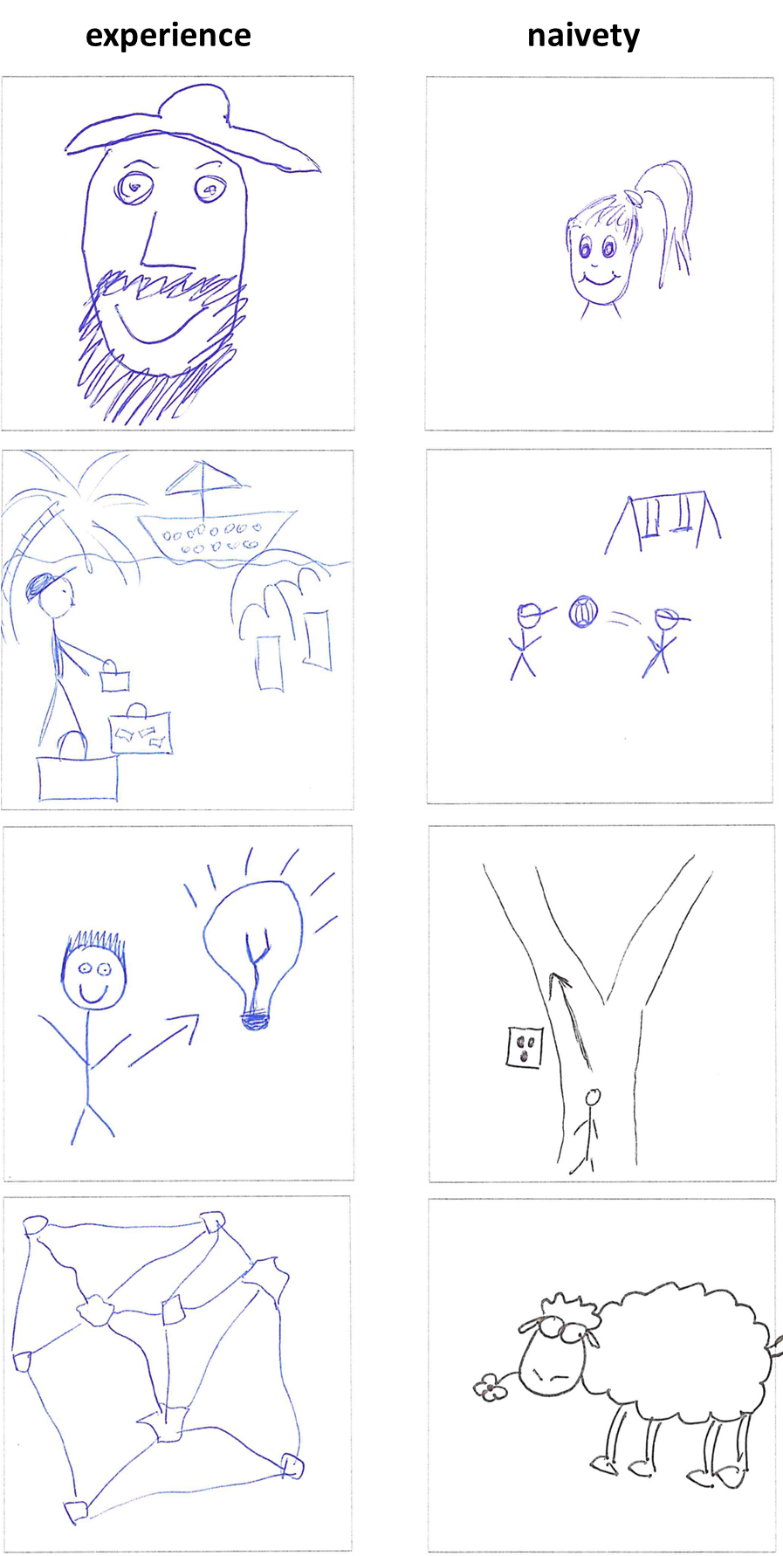
*

**Wisdom/Foolishness**

In Figure SM5 samples of participants’ drawings for the concept pair wisdom/foolishness are depicted. Drawings indicated that that wisdom was associated with older age and the male sex. Foolishness was not systematically associated with any sex. For wisdom, often books were drawn, sometimes an owl. For wisdom, sometimes some form of knowledge transfer was implied, for instance between grown up-ups and children. For stupidity, a lack of brain or being puzzled was often implied. One recurring theme in foolishness was drugs (either as objects or as the action of taking drugs or drunk driving).

**Figure S4**

*Samples of Participants’ Drawings for the Concept Pair Wisdom/Foolishness*


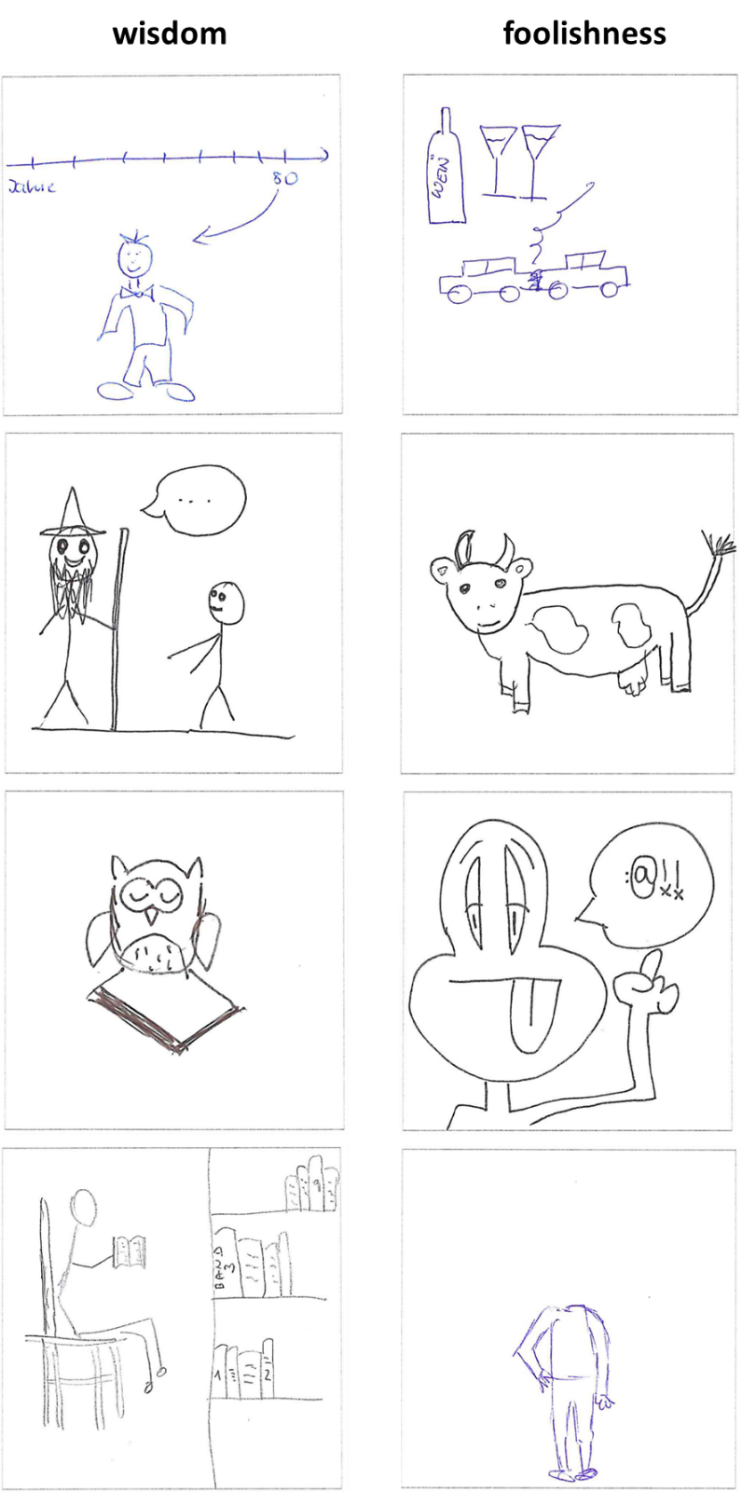

Supplement: Supplementary file 1 — (DOCX 2066 kb) [file 13421_2023_1492_MOESM1_ESM.docx]
